# Integrative Genomics and Multi-Tissue Transcriptomics Identify Key Loci and Pathways for Hypoxia Tolerance in Grass Carp

**DOI:** 10.3390/ani15243518

**Published:** 2025-12-05

**Authors:** Wenwen Wang, Mengyang Chang, Suxu Tan, Yiming Hu, Xinlu Ren, Hongtao Xue, Lizheng Gao, Xiao Cao, Ya Wang, Qiyu Li, Zhenxia Sha

**Affiliations:** 1 Institute of Aquatic Biotechnology, College of Life Sciences, Qingdao University, Qingdao 266071, China; 2 Shandong Center of Technology Innovation for Biological Breeding of Premium Fish (Preparatory), Yantai 261418, China; 3 State Key Laboratory of Mariculture Biobreeding and Sustainable Goods, Yellow Sea Fisheries Research Institute, Chinese Academy of Fishery Sciences, Qingdao 266071, China; 4 Weishan County Nansihu Fishery Co., Ltd., Jining 277600, China; 5 Laboratory for Marine Fisheries Science and Food Production Processes, Qingdao Marine Science and Technology Center, Qingdao 266237, China

**Keywords:** grass carp, hypoxia, GWAS, RNA-seq, SNPs

## Abstract

Hypoxia is a critical environmental stressor in aquaculture, significantly affecting the survival and growth performance of cultured fish. To explore the genetic basis of hypoxia tolerance in grass carp (*Ctenopharyngodon idella*), we integrated genome-wide association analysis (GWAS) and multi-tissue transcriptome profiling. A total of 2000 grass carp were subjected to hypoxic stress, from which the 150 most hypoxia-intolerant (HI) and 150 most hypoxia-tolerant (HT) individuals were selected based on the time to loss of equilibrium (LOE). GWAS using 3,730,919 SNPs and 851,595 InDels identified 21 SNPs and 6 InDels associated with hypoxia tolerance. Two SNPs on chromosomes 10 and 13 reached genome-wide significance, accounting for 2.7% and 4.8% of the phenotypic variance explained (PVE), respectively. Validation of identified SNPs was performed using kompetitive allele-specific PCR (KASP) analysis. Candidate genes within ±50 kb of these variants were enriched in steroid biosynthesis, insulin signaling, and glycosphingolipid biosynthesis pathways. Transcriptomic analysis of six tissues (brain, gill, intestine, kidney, liver, and spleen) revealed 1620, 1221, 796, 246, 210, and 58 differentially expressed genes (DEGs) in the HT group compared to the HI group, respectively. DEGs in the brain were primarily enriched in steroid metabolic processes and angiogenesis regulation, while those in kidney and spleen DEGs were associated with oxygen transport and erythrocyte development. Integrated analysis of GWAS and transcriptome data identified 16 shared genes, including *usf1* and *trpv4*. These findings reveal key genomic loci and molecular pathways underlying hypoxia tolerance in grass carp, providing valuable markers for future selective breeding programs.

## Simple Summary

Hypoxia is a major threat to the survival and performance of cultured fish. In this study, we combined genome-wide association analysis (GWAS) with multi-tissue transcriptome profiling to investigate the genetic basis of hypoxia tolerance in grass carp. We identified 21 SNPs, 6 InDels, and 16 candidate genes, including *usf1* and *trpv4*, associated with hypoxia response. These results provide useful genomic markers and resources to support selective breeding for improved hypoxia tolerance in grass carp.

## 1. Introduction

Hypoxia, a condition characterized by reduced dissolved oxygen (DO) levels in water, is a major environmental stressor affecting aquatic ecosystems worldwide [1]. In natural habitats, hypoxia can result from eutrophication, algal blooms, and thermal stratification [1]. In aquaculture systems, hypoxia mainly occurs under the following conditions: rainy/overcast days, high-temperature weather, the period from night-time to early morning, excessively high stocking density, or deterioration of water quality [2]. Hypoxia can cause metabolic disorders, weakened immunity, slow growth, decreased reproduction, and even death in fish [3]. Teleost fish are good models for hypoxia studies because they inhabit aquatic habitats with varying oxygen levels, and they have evolved a suite of physiological and biochemical adaptations, including metabolic suppression, enhanced anaerobic glycolysis, and increased oxygen uptake efficiency [4,5]. Different fish species, and even individuals within the same species, show considerable variation in hypoxia tolerance [6,7]. The genetic basis underlying this interspecific and intraspecific variation in hypoxia tolerance is complex and continues to be a key area of research.

Genome-wide association study (GWAS) has successfully identified genetic markers for disease resistance, growth, and stress tolerance in various fish species [8,9]. Multiple GWAS have identified genetic variants linked to fish hypoxia tolerance. For example, in a study on large yellow croaker (*Larimichthys crocea*), seven significant hypoxia-associated single nucleotide polymorphisms (SNPs) were identified by GWAS, and the shared genes from GWAS and differentially expressed genes (DEGs) from transcriptome analysis were related to glucose transport and metabolism, erythropoiesis, ion regulation, DNA replication, and repair [10]. In hybrid catfish, four quantitative trait loci (QTL) were found to be associated with low oxygen tolerance by GWAS using the catfish 250 K SNP array [11]. For channel catfish (*Ictalurus punctatus*) under hypoxia stress, one significant QTL across strains and six significant QTL within strains were identified and candidate genes were predicted to function in oxygen metabolism and to participate in MAPK or PI3K/AKT/mTOR signaling pathways [12]. A total of 4 significant SNPs and 16 potential candidate genes were identified as being associated with hypoxia tolerance in golden pompano (*Trachinotus ovatus*) [13]. However, research on fish hypoxia tolerance using GWAS remains relatively limited, with even fewer applications in molecular breeding.

Grass carp (*Ctenopharyngodon idella*), an economically important native Chinese freshwater fish, has been widely cultivated worldwide. Grass carp is the fish species with the highest aquaculture output in China and has contributed significantly to food security and rural economies in Asia [14,15]. Currently, research on grass carp breeding primarily focuses on traits such as growth and disease resistance, as well as GWAS on genetic loci associated with these traits [16,17]. With the development of intensive farming practices, stress-resistant traits, such as hypoxia tolerance and ammonia tolerance, have gained increasing attention [18]. In this study, we employed a combined GWAS and transcriptomic approach to identify SNPs, insertions and deletions (InDels), and potential candidate genes associated with hypoxia tolerance in grass carp, offering molecular markers for breeding programs and advancing our understanding of adaptive responses to environmental stress in aquatic species.

## 2. Materials and Methods

### 2.1. Animal Welfare Statement

All handling of the fish was performed in accordance with the Guidelines for the Care and Use of Laboratory Animals of China and approved by the Ethics Committee of the Medical College of Qingdao University, China (QDU-AEC-2024004).

### 2.2. Hypoxia Treatment and Sample Collection

The experiment was conducted at Weishan County Nansihu Fishery Co., Ltd., Jining, China. The grass carp cultivated by this company originates from fry procured from a hatchery in Guangdong Province (multiple breeding families and non-random mating). In this study, a total of 2000 six-month-old grass carp with an average body weight of 23.50 g and body length of 12.58 cm were collected and distributed into five 150 L tanks (each containing 120 L of water). The fish were acclimated for one week in tanks at a water temperature of 21.0 ± 1.0 °C and a DO level of 6.0 ± 0.5 mg/L by aeration. During the hypoxic stress experiment, the aeration was stopped and DO level was monitored by a water quality meter (YSI ProQuatro, Yellow Springs, OH, USA). According to the previous studies, DO levels were decreased by adding sodium sulfite [11,12,19,20]. The water temperature, pH, and ammonia nitrogen were monitored every 2 h to ensure stable experimental conditions.

The time interval from when DO dropped to 0.1 mg/L until each fish exhibited loss of equilibrium (LOE) was recorded as the hypoxia-tolerance phenotype. After reaching LOE, fish were euthanized with tricaine methanesulfonate (MS-222), and body weight and body length were measured. Fin clips were collected from the first 150 fish that reached LOE (hypoxia-intolerant fish, HI) and the last 150 fish (hypoxia-tolerant fish, HT) for genomic DNA extraction. In addition, tissues including brain, gill, intestine, kidney, liver, and spleen from the first three HI fish and the last three HT fish were collected for total RNA extraction, resulting in 36 tissue samples (3 replicates × 2 groups × 6 tissues). Each collected sample was placed in a cryogenic vial, immediately frozen in liquid nitrogen, and stored at −80 °C for subsequent analysis.

### 2.3. Genomic DNA Extraction and Sequencing

Genomic DNA was extracted from 300 fin samples using the PureLink Fast DNA Tissue Kit (Invitrogen, Carlsbad, CA, USA), following the manufacturer’s protocol. The DNA concentration and quality were assessed using a nucleic acid analyzer (OSTC, Beijing, China). High-quality genomic DNA was stored at −20 °C for subsequent library construction. The average DNA concentration was 80–100 ng/μL, and the OD260/280 ratios ranged from 1.8 to 2.0. Libraries were constructed with the NEBNext^®^ UltraTM DNA Library Prep Kit (NEB, Ipswich, MA, USA). Following quality control, paired-end libraries with 350 bp insert size were constructed. The DNA libraries were sequenced on the DNBSEQ-T7 platform (MGI Tech, Shenzhen, China), generating paired-end 150 bp reads with an average depth of ~10× per sample.

### 2.4. Genotyping and Filtering

The raw DNA sequencing data were assessed for quality and filtered using fastp software (version 0.23.4) [21] to remove adapter contamination and low-quality reads. After filtering, high-quality clean reads were generated and subsequently mapped to the genome of the *C. idella* (GCA_019924925.1) [22] using BWA software (version 0.7.17) [23]. Duplicate reads were marked and removed using Picard Tools (version 2.25.0) (https://github.com/broadinstitute/picard, 4 April 2025). Variant calling was performed to identify SNPs and InDels variants using GATK (version 4.5.0.0) [24]. SNPs were subjected to hard filtering with the following parameters: QualByDepth (QD) < 2.0, FisherStrand (FS) > 60.0, RMSMappingQuality (MQ) < 40.0, ReadPosRankSumTest (ReadPosRankSum) < −8.0, MappingQualityRankSumTest (MQRankSum) < −12.5, and SOR ≥ 3.0. For InDel filtering, the parameters were: QD < 2.0, MQRankSum < −12.5, FS > 200.0, ReadPosRankSum < −8.0, and SOR > 10.0. After variant filtering, PLINK software (version 1.9) [25] was used for additional quality control with the following thresholds: (1) missing genotype rate per SNP (-geno) < 0.01, (2) individual missing rate (-mind) > 0.02, (3) minor allele frequency (-maf) < 0.05, and (4) Hardy–Weinberg equilibrium (-hwe) < 0.001. To categorize the functional effects of the variants, SnpEff software (version 5.0) was utilized for annotation based on the annotated genome of *C. idella*.

### 2.5. Linkage Disequilibrium (LD) and Population Structure Analyses

Only autosomal SNPs with MAF > 0.05 were retained for LD and population structure analyses. LD analysis was conducted using PopLDdecay software (version 3.42), which calculates the LD coefficient (r^2^) between pairs of variants (SNPs and InDels) [26]. The genome-wide LD decay pattern as a function of physical distance was visualized using the Plot_OnePop Perl script provided with the PopLDdecay package. To investigate potential genetic relatedness within the population, population structure analysis was performed. Principal component analysis (PCA) was conducted using PLINK (version 1.9) [25], and the first two principal components (PC1 and PC2) were plotted to visualize sample clustering. Additionally, Admixture software (version 1.3.0) [27] was used to estimate population structure. The number of ancestral populations (K) was set from 1 to 15, and the optimal K value was determined based on the minimum cross-validation (CV) error. Visualization of the population structure results was performed using the R package ggplot2 (version 4.2.1).

### 2.6. Genome-Wide Association Study (GWAS)

GWAS was conducted using GEMMA software (version 0.98.5) based on a linear mixed model (LMM) [28]. First, the genetic kinship between individuals was estimated using the parameter: -gk 2. The resulting genetic relatedness matrix was then applied in the GWAS analysis, with model adjustments made based on the results from PCA. The GWAS model used is expressed as follows:

y = Wα + Xβ + Zμ + ϵ

where y is the phenotype vector, W is the covariate matrix (including intercept and PC covariates), α is the vector of fixed effects, X is the genotype matrix, β is the marker effect, Z is the random effect design matrix, μ is the random effect vector, and ϵ is the residual.

To account for multiple testing, we applied the Bonferroni correction method, setting the significance threshold at 0.05/N for significant associations and 1/N for suggestive associations, where N represents the total number of variants analyzed. Manhattan and quantile-quantile (Q-Q) plots were generated using the CMplot package (version 4.5.10) to visualize the GWAS results. The phenotypic variance explained (PVE) value of the variants was calculated based on the published formula. The SnpEff software (version 5.0) [29] was used to construct the genome annotation database of *C. idella* using genome annotation files and reference genome sequences. Subsequently, the identified SNPs from GWAS were annotated. Considering the genome-level LD in *C. idella*, genomic regions flanking the detected SNPs (50 kb upstream and downstream) were identified for candidate genes. Kyoto Encyclopedia of Genes and Genomes (KEGG) pathway analyses of candidate genes were performed using the clusterProfiler package (version 4.6.2) [30] in R. Protein–protein interaction (PPI) network of candidate genes was constructed using String (https://cn.string-db.org/, 4 April 2025).

### 2.7. Total RNA Extraction, Transcriptome Sequencing and Data Processing

Total RNA was extracted from 36 samples, including brain, gill, intestine, kidney, liver, and spleen from three HI fish and three HT fish using TRIzol reagent (Qiagen, Hilden, Germany). RNA integrity was assessed using Agilent 2100 Bioanalyzer (Agilent Technologies, Santa Clara, CA, USA), and samples with RNA Integrity Number (RIN) ≥ 7.0 were used for library preparation. The sequencing libraries were constructed using Stranded RNA LibraryPrep Kit (lllumina, San Diego, CA, USA). The libraries were sequenced on the DNBSEQ-T7 platform (MGI Tech, Shenzhen, China), generating paired-end 150 bp reads.

The raw reads in FASTQ format were initially processed using fastp [21], which removed low-quality reads to generate clean reads. These clean reads were subsequently aligned to the reference genome (GCA_019924925.1) using HISAT2 (version 2.0.5) [31]. Gene expression levels were quantified as Fragments Per Kilobase of exon model per Million (FPKM), and read counts were obtained using featureCount (version 2.0.4) [32]. PCA was conducted in R (version 4.2) to assess the biological duplication of samples. Differential expression analysis was performed using DESeq2 (version 1.40.2) [33], with a threshold of adjust *p*-value < 0.05 and fold change > 2 to identify DEGs. Hierarchical clustering analysis of DEGs was performed to illustrate expression patterns across different groups and samples using R (version 4.2). A radar map to visualize the expression of upregulated or downregulated DEGs was generated using the ggplot2 package in R (version 4.2). Furthermore, Gene Ontology (GO) and KEGG pathway analyses of DEGs were carried out using the clusterProfiler (version 4.6.2) [30] package in R to elucidate the functional roles of the identified DEGs.

### 2.8. Validation of Significant SNPs

In this study, two SNPs [(the SNP at position 33,445,030 bp on chromosome 5 (SNP7) and the SNP at position 36,269,337 bp on chromosome 10 (SNP11)] that showed strong statistical associations with hypoxia tolerance based on GWAS and subsequent filtering criteria were selected for KASP genotyping. A total of 300 individuals were used to validate the genotype–phenotype relationship of these loci. Each SNP genotype was called using fluorescence signal clustering in the Bio-Rad CFX96 system, and the accuracy was verified by randomly re-genotyping 10% of the samples. The primers were designed on https://tools.goodbtk.com/SNPPrimer (20 April 2025) and listed in Supplementary Table S1. PCR reactions were conducted with a final volume of 2 μL in each well, containing 1 μL Flu-Arms 2× PCR mix V4 (GoodBTK, Guangzhou, China), 0.02 μL primer F1 (10 μM), 0.02 μL primer F2 (10 μM), 0.06 μL primer R (10 μM), DNA template (5–50 ng), and water to a total volume of 2 μL. Genotype-phenotype association was re-evaluated using Chi-square test between allelic groups.

## 3. Results

### 3.1. Phenotype Statistics

**Figure 1:**
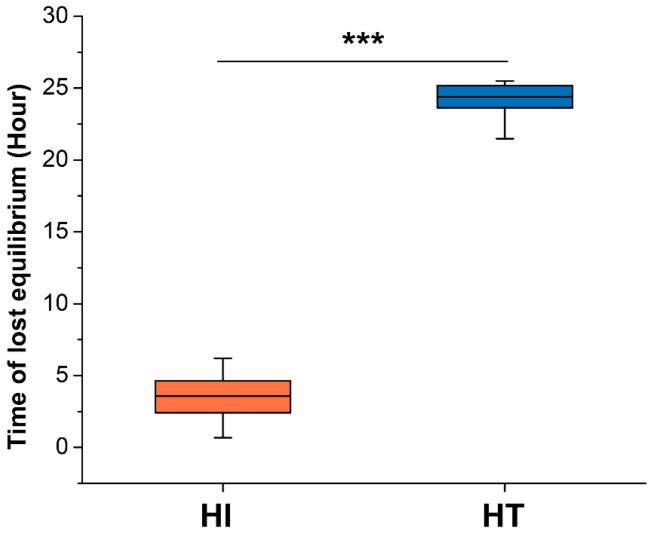
Comparison of lost equilibrium time in the hypoxia-intolerant (HI) and hypoxia-tolerant groups (HT). Asterisk (***) indicates statistical significance between HI and HT groups using Welch’s *t*-test, *** *p* < 0.001.

The first grass carp to lose equilibrium was observed at 0.68 h after the addition of sodium sulfite. As the duration of hypoxia stress increased, individual fish successively lost equilibrium. The average time for the first 150 fish to lose balance was 3.49 h, while that for the latter 150 fish was 23.62 h (Figure 1). Notably, a significant difference (*p* =7.65 × 10^−192^) was detected in the time to LOE between the HI and HT groups (Figure 1), highlighting marked inter-individual variation in the hypoxia tolerance trait of grass carp.

### 3.2. Genotyping and Population Structure

**Figure 2:**
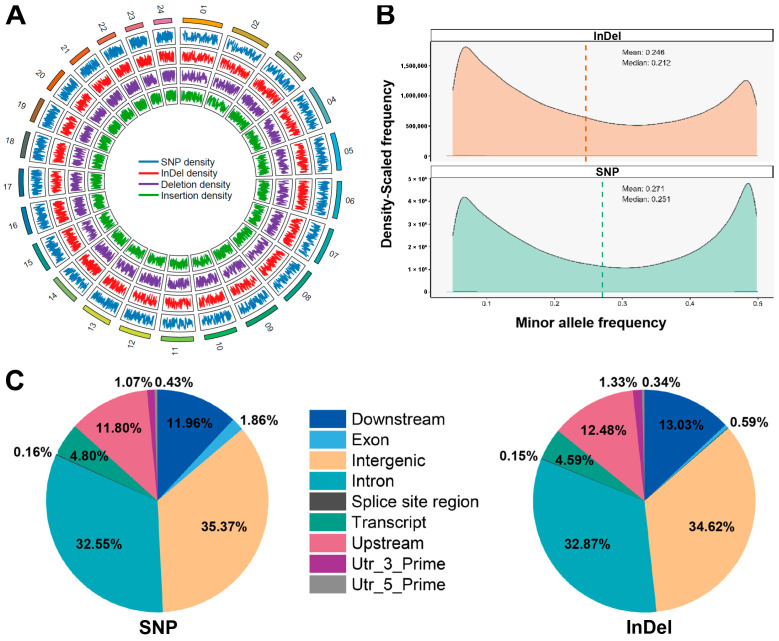
The characteristics of SNPs and InDels in grass carp. (**A**) Density and distribution of SNPs and InDels among 24 chromosomes. From outermost to innermost, the tracks represent: chromosomes, SNP density, InDel density, deletion density, and insertion density. (**B**) The minor allele frequency (MAF) distribution of SNPs and InDels. (**C**) Frequency of genomic region positions for SNPs and InDels.

After genotyping and quality filtering, a total of 3,730,919 SNPs and 851,595 InDels were identified across 300 fish. These SNPs and InDels were widely distributed across the 24 chromosomes (Figure 2A). Based on the 893.2-Mb reference genome, the combined variants correspond to an average density of approximately 5.13 markers per kb, indicating a generally uniform genome-wide distribution without strong clustering signals. The MAF distributions of SNPs and InDels are shown in Figure 2B. The genome-wide average density rate was calculated as 243 bp/SNP and 963 bp/InDel. Of these variants, 1,214,563 (32.55%) SNPs and 279,885 (32.87%) InDels were located in introns, 1,319,775 (35.37%) SNPs and 294,830 (34.62%) InDels were identified in intergenic regions, and 69,245 (1.86%) SNPs and 5007 (0.59%) InDels were located in exons (Figure 2C).

**Figure 3:**
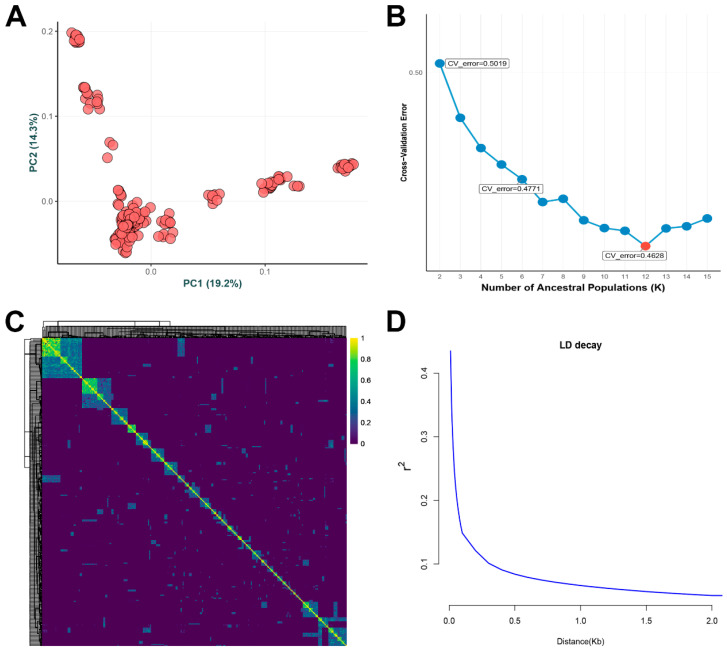
Population structure analyses using SNPs of grass carp. (**A**) Principal component analysis (PCA) analysis. (**B**) Variation in cross-validation (CV) error at different K values. (**C**) Heatmap of genomic relatedness. (**D**) Linkage disequilibrium (LD) decay.

The PCA using high-quality SNPs revealed that the grass carp used in this study belonged to several discrete sub-populations (Figure 3A). When K = 12, the CV value reached its minimum (Figure 3B), indicating this was the optimal model for describing the population genetic structure. This suggests that the population consisted of 12 genetically distinct sub-populations. In addition, the genetic relatedness matrix showed weak genetic relatedness among the grass carp used in this study (Figure 3C), reflecting a high level of genetic diversity within the population. The LD analysis revealed a maximum r^2^ value of 0.44, followed by a sharp decrease to 0.1 at a distance of 400 bp (Figure 3D). Population structure analysis, including PCA (Supplementary Figure S1A) and genetic relatedness matrix (Supplementary Figure S1B) using InDels, was also conducted, supporting the findings from the SNP analysis.

### 3.3. GWAS

GWAS for the HI and HT groups was conducted using 150 genotyped samples each. Association analyses of HI and HT traits were performed using LMM with 3,730,919 SNPs and 851,595 InDels. The threshold *p* value for genome-wide statistical significance was 0.05/3,730,919 = 1.34 × 10^−8^ [−log_10_ (*p* value) = 7.87] for SNPs. The threshold *p* value for the suggestive association was 1/3,730,919 = 2.68 × 10^−7^ [−log_10_ (*p* value) = 6.57] for SNPs. For the InDels, the significant and suggestive thresholds were −log_10_ (0.05/851,595) = 7.23 and −log_10_ (1/851,595) = 5.93, respectively.

**Figure 4:**
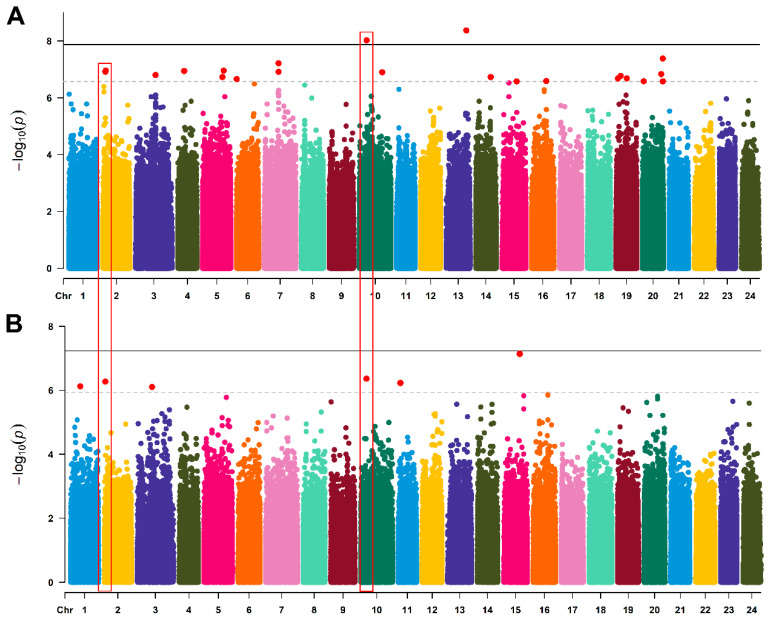
Manhattan plots of genome-wide association analysis for hypoxia tolerance in grass carp. (**A**) Manhattan plot for SNPs. (**B**) Manhattan plot for InDels. The solid line indicates the threshold for significance, and the dotted line indicates the threshold for suggestive association. The red boxes represent the QTL shared between the GWAS results of SNPs and InDels.

The Manhattan plots of the GWAS results are shown in Figure 4. A total of 21 SNPs were identified in association with hypoxia tolerance in grass carp, located on chromosome 2, 3, 4, 5, 6, 7, 10, 13, 14, 16, 19 and 20 (Table 1, Figure 4A). Two SNPs on chromosome 10 and 13 were significantly associated with hypoxia tolerance with PVE values of 4.8% and 2.7%, respectively (Table 1). A total of 6 suggestive InDels were detected on chromosome 1, 2, 3, 10, 11 and 15 (Figure 4B). The QTL shared between the GWAS results of SNPs and InDels were located on chromosome 2 and 10. The Quantile-quantile (Q-Q) plots confirmed the reliability and validity of our GWAS analysis (Supplementary Figure S2), demonstrating that the statistical model was appropriately specified for this study.

### 3.4. Genes Within the QTL Regions

**Figure 5:**
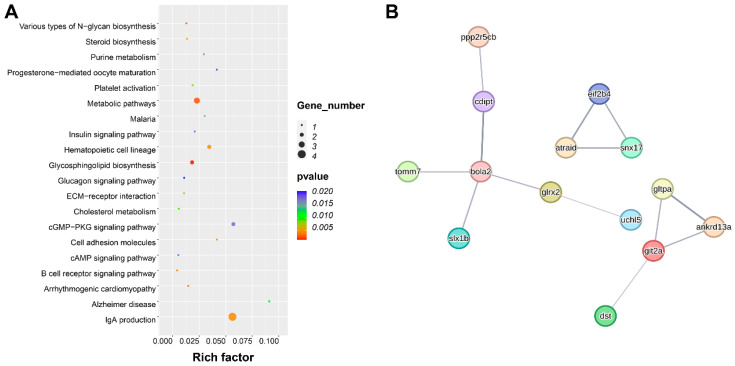
Analysis of candidate genes associated with SNP and InDels. (**A**) KEGG enrichment analysis of candidate genes. (**B**) Results of protein–protein interaction network (PPI) analysis of candidate genes.

To provide insights into the potential candidate genes associated with hypoxia tolerance, we examined the ±50 kb genomic regions surrounding the significant and suggestive SNPs and InDels. A total of 89 genes were identified in the SNP-associated regions (Supplementary Table S2) and 34 genes in the InDel-associated regions (Supplementary Table S3). To further investigate their functional relevance, KEGG pathway enrichment analysis was performed (Figure 5A). These candidate genes were significantly enriched in pathways such as steroid biosynthesis, insulin signaling, glycosphingolipid biosynthesis, IgA production, and glucagon signaling.

Furthermore, we constructed a PPI network to identify potential hub genes (Figure 5B). Fourteen key candidate genes were highlighted, including protein phosphatase 2, regulatory subunit B, gamma b (*ppp2r5cb*), CDP-diacylglycerol--inositol 3-phosphatidyltransferase (*cdipt*), Zgc: 112,271 protein (*bola2*), translocase of outer mitochondrial membrane 7 homolog (*tomm7*), SLX1 homolog B, structure-specific endonuclease subunit (*slx1b*), glutaredoxin 2 (*glrx2*), ubiquitin carboxyl-terminal hydrolase L5 (*uchl5*), dystonin (*dst*), G protein-coupled receptor kinase interacting ArfGAP 2a (*git2a*), glycolipid transfer protein a (*gltpa*), ankyrin repeat domain 13A (*ankrd13a*), all-trans retinoic acid-induced differentiation factor (*atraid*), sorting nexin 17 (*snx17*), and eukaryotic translation initiation factor 2B, subunit 4 delta (*eif2b4*). These genes may play central roles in the regulation of hypoxia tolerance in grass carp.

### 3.5. Differential Expression Between HI and HT Groups

RNA-seq was conducted in brain, intestine, kidney, liver, gill, and spleen for both the HI and HT groups. A total of 1791 million raw reads were produced, with 1710 million clean reads retained after quality filtering (Supplementary Table S4). The raw data have been submitted to NCBI SRA with the BioProject ID PRJNA1306682. The average mapping rate was 91.98% across all samples (Supplementary Table S4). Comparative analysis of expression levels between HI and HT groups revealed tissue-specific differential gene expression: 1620, 1221, 796, 246, 210, and 58 DEGs were identified in the brain, intestine, kidney, liver, gill, and spleen, respectively (Supplementary Figure S3).

**Figure 6:**
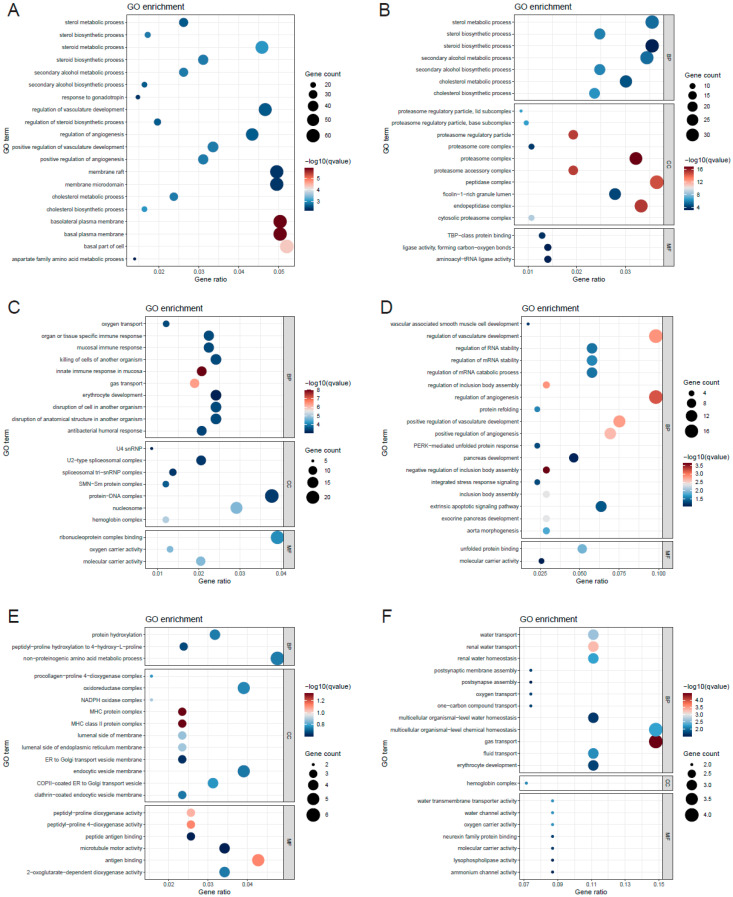
GO enrichment analysis of DEGs between HI and HT groups in different tissues: (**A**) brain; (**B**) intestine; (**C**) kidney; (**D**) liver; (**E**) gill; (**F**) spleen. Gene Ontology (GO) terms are categorized into three domains: BP (Biological Process), representing biological objectives or processes; CC (Cellular Component), referring to the cellular locations where gene products function; and MF (Molecular Function), describing the biochemical activities of gene products.

GO analysis revealed that DEGs in brain tissue were significantly enriched in biological processes (BP) such as steroid metabolic process, cholesterol biosynthetic process, cholesterol metabolic process, positive regulation of angiogenesis, and positive regulation of blood vessel development (Figure 6A). In the intestine, GO terms consistently enriched with those in the brain included steroid metabolic process, sterol biosynthetic process, cholesterol metabolic process, and related terms. Furthermore, DEGs were also enriched in tRNA metabolic process, tRNA aminoacylation for protein translation, tRNA aminoacylation, outer mitochondrial membrane organization, etc. (Figure 6B). DEGs in the kidney were enriched in innate immune response in mucosa gas transport, mucosal immune response, oxygen transport, erythrocyte development/differentiation and heme metabolic process (Figure 6C). The enriched GO terms included negative regulation of inclusion body assembly, regulation of angiogenesis, regulation of inclusion body assembly, regulation of vasculature development, positive regulation of angiogenesis, etc. in the liver (Figure 6D). GO BP terms, such as protein hydroxylation, peptidyl-proline hydroxylation to 4-hydroxy-L-proline, non-proteinogenic amino acid metabolic process, were enriched in the gill (Figure 6E). In addition, DEGs in the spleen were enriched in gas transport, oxygen transport, one-carbon compound transport, postsynaptic membrane assembly and erythrocyte development (Figure 6F).

**Figure 7:**
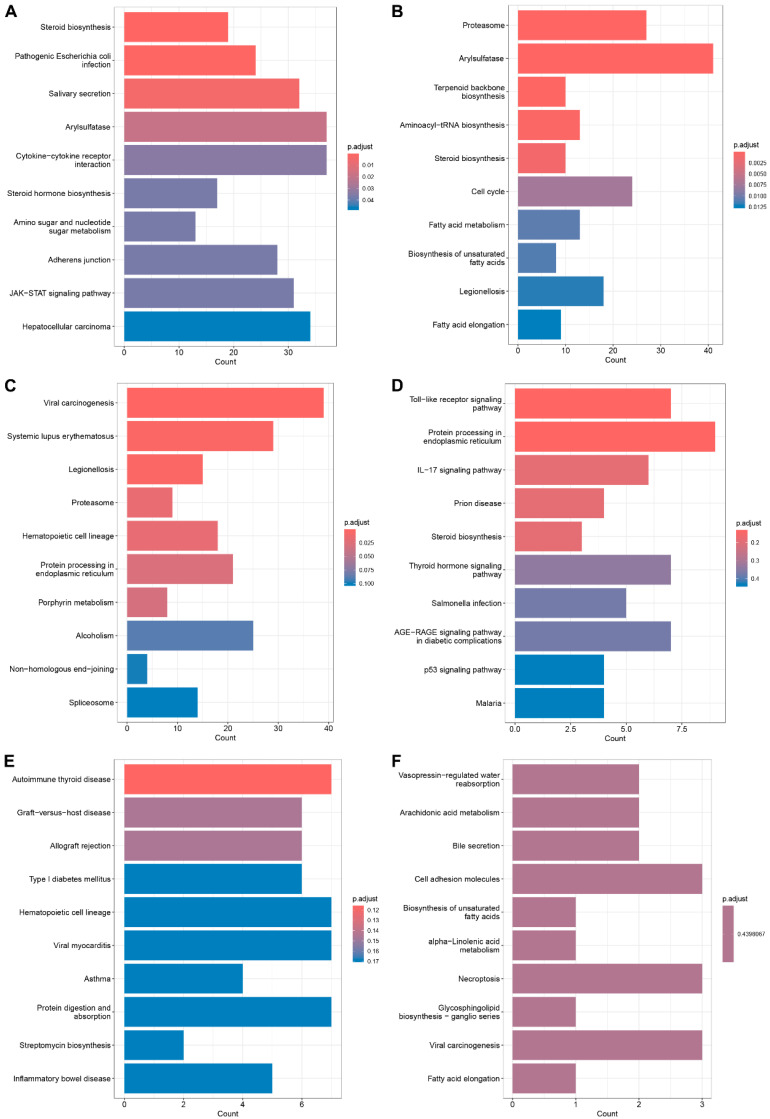
KEGG analysis of DEGs between HI and HT groups in different tissues: (**A**) brain; (**B**) intestine; (**C**) kidney; (**D**) liver; (**E**) gill; (**F**) spleen.

KEGG analysis of DEGs in different tissues was carried out (Figure 7). The enriched KEGG pathways were as follows: in the brain (Figure 7A), they included steroid biosynthesis, cytokine-cytokine receptor interaction, steroid hormone biosynthesis, amino sugar and nucleotide sugar metabolism, and the JAK-STAT signaling pathway; in the intestine (Figure 7B), they were proteasome, terpenoid backbone biosynthesis, aminoacyl-tRNA biosynthesis, steroid biosynthesis, and amino sugar and nucleotide sugar metabolism; in the kidney (Figure 7C), they were proteasome, hematopoietic cell lineage, protein processing in the endoplasmic reticulum, and porphyrin metabolism; in the liver (Figure 7D), they were Toll-like receptor signaling pathway, protein processing in the endoplasmic reticulum, IL-17 signaling pathway, Steroid biosynthesis, and Thyroid hormone signaling pathway; in the gill (Figure 7E), they were autoimmune thyroid disease, hematopoietic cell lineage, and viral myocarditis; in the spleen (Figure 7F), they were vasopressin-regulated water reabsorption, arachidonic acid metabolism, cell adhesion molecules, biosynthesis of unsaturated fatty acids, and alpha-Linolenic acid metabolism.

### 3.6. Joint Analysis of GWAS and RNA-Seq

To identify candidate genes associated with hypoxia tolerance in grass carp, we determined 16 shared genes between the potential candidate genes identified by GWAS and DEGs obtained by RNA-seq analysis (Table 2). Eight, four, four, and two genes showed differential expression in the brain, kidney, intestine, and liver, respectively. Notably, among them, upstream transcription factor 1 (*usf1*) was annotated for its function in “response to hypoxia”, and transient receptor potential cation channel, subfamily V, member 4 (*trpv4*) for its “ATP binding” function.

### 3.7. Validation of SNP Genotype Frequency by qPCR

**Figure 8:**
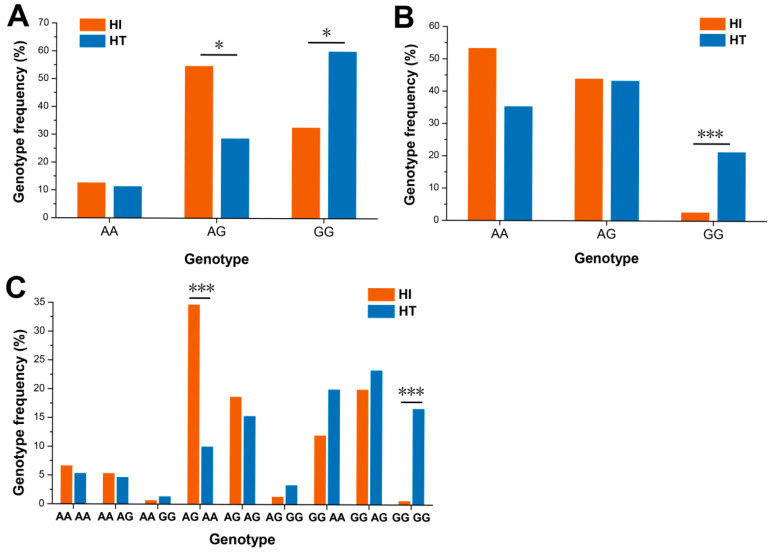
The genotype frequency of the identified SNPs in the hypoxia intolerance (HI) and hypoxia tolerance (HT) groups. (**A**) The genotype frequencies of SNP7, the SNP at position 33,445,030 bp on chr 5. (**B**) The genotype frequencies of SNP11, the SNP at position 36,269,337 bp on chr 10. (**C**) The genotype combination frequencies for SNP7 and SNP11 (with the order being SNP7 followed by SNP11). Asterisk (*) indicates statistical significance between HI and HT groups using Chi-square test, * *p* < 0.05, *** *p* < 0.001.

Two statistically significant SNPs identified in the GWAS analysis—the SNP located at 33,445,030 bp on chromosome 5 (SNP7) and the SNP located at 36,269,337 bp on chromosome 10 (SNP11)—were selected for KASP genotyping. Both SNPs showed a strong association between genotype and hypoxia tolerance, with individuals carrying the GG genotype exhibiting significantly higher tolerance compared with those carrying AG or AA genotypes (Figure 8A,B). For both loci, GG/GG represented the superior genotype, whereas AG/AA corresponded to inferior hypoxia-tolerance performance (Figure 8C).

## 4. Discussion

Given the widespread adoption of high-density aquaculture and the increasing prevalence of hypoxic waters globally [34,35,36], aquatic hypoxia has become a pervasive challenge for various fish populations, including marine fish, freshwater fish, aquaculture fish, and wild fish, and dramatically impacts fisheries and aquaculture worldwide. To date, there are no GWAS researches on hypoxia tolerance in grass carp. For the first time, we used GWAS to identify SNPs and InDels associated with hypoxia tolerance in grass carp. Considering that the population structure is characterized by non-random mating and multiple breeding families, we took this into account during the analysis to ensure the reliability of the associations. In combination with transcriptome analysis, potential candidate genes were further identified. This study provided novel insights into the adaptive strategies of grass carp under hypoxia stress and offered a theoretical basis and genetic markers for molecular breeding.

The time to LOE, a widely used indicator of hypoxia tolerance in fish [12,37,38], showed a striking difference between HT and HI groups of grass carp, with average values of 23.62 h and 3.49 h, respectively. This marked variation suggested the presence of heritable genetic components underlying hypoxia tolerance in grass carp, consistent with observations in other fish species such as channel catfish [12,20] and hybrid catfish [11]. Such phenotypic divergence provides a solid foundation for GWAS and genomic selecting breeding.

From the GWAS results, 21 SNPs and six InDels associated with hypoxia tolerance, distributed across 12 chromosomes were identified, indicating that hypoxia tolerance trait in grass carp are regulated by micro-efficient multiple genetic loci. The low individual phenotypic variance explained (PVE: 4.8% and 2.7%) by the identified two significant SNPs aligns with GWAS findings for the polygenic architecture of complex traits, where minor-effect loci collectively drive phenotypic variation. This reflects the trait’s inherent polygenicity, alongside potential limitations in variation coverage or sample size that limit detection of small-effect loci. Notably, these SNPs remain biologically relevant, as they were localized to hypoxia-responsive genes and they are potentially correlated with gene expression. Future studies with larger samples or multi-omics integration will help uncover additional loci and enhance cumulative PVE, advancing understanding of the trait’s genetic basis. Overlapping QTL regions on chromosomes 2 and 10 were detected for both SNPs and InDels, suggesting these regions may harbor conserved genetic elements regulating hypoxia responses. The candidate genes within ± 50 kb of these variants were enriched in pathways such as steroid biosynthesis, insulin signaling, and glycosphingolipid biosynthesis. Steroid biosynthesis, in particular, was consistently highlighted in both GWAS and transcriptome analyses. Previous researches have already demonstrated that hypoxia affects steroid biosynthesis [39,40]. Steroids, including corticosteroids, are known to modulate metabolic and stress responses in fish [41]. Hypoxia-inducible factor 1 (HIF1) is an oxygen-regulated transcriptional activator [42], and plays a significant role in the production of steroids [39].

Transcriptome profiling revealed distinct DEG patterns across six tissues, reflecting specialized roles in hypoxia adaptation. The most DEGs (1620) were identified in the brain, enriched in steroid metabolic processes and angiogenesis regulation. Steroids may modulate neural activity and energy metabolism in the brain, a tissue highly sensitive to oxygen deprivation. Enhanced angiogenesis (e.g., via upregulated genes in “positive regulation of blood vessel development”) could improve oxygen delivery to neurons, a strategy observed in hypoxia-tolerant fish [43]. DEGs in these six tissues were enriched in oxygen transport and erythrocyte development, indicating enhanced hematopoiesis and oxygen-carrying capacity in HT fish. This aligns with the well-known role of the HIF pathway in promoting erythropoiesis [44] and suggests that HT fish may optimize oxygen transport to tissues. Enrichment of steroid biosynthesis and proteasome pathways in the liver and intestine highlights their roles in metabolic adjustment and protein turnover. The liver, a central metabolic organ, may shift to anaerobic metabolism under hypoxia [45].

The PPI network analysis of the GWAS-identified genes revealed 14 key genes, such as *cdipt* and *uchl5*. Among these genes, *cdipt*, which encodes a key enzyme in phosphatidylinositol synthesis, was significantly reduced in mice after 12 h and 24 h of hypobaric hypoxia exposure [46]. This finding demonstrates the involvement of lipid metabolism in the hypoxic stress response and highlights the critical role of this gene. Furthermore, its down-regulation in the liver of common carp (*Cyprinus carpio* L.) under high-temperature stress suggested a conserved role for *cdipt* in responding to diverse environmental stresses [47]. This study found that HIF-1α transcriptionally activates *uchl5* expression [48]. Given that HIF1 is an oxygen-regulated transcription activator, this result demonstrated that *uchl5* has a specific function in hypoxic stress. Collectively, these results provide a foundation for further studies on the roles of these genes in environmental stress responses in fish.

The 16 genes shared between GWAS candidates and DEGs represent high-priority targets for functional validation in subsequent studies. The functional association between the validated SNPs and gene expression remains elusive. Demonstrating causality would require further verification, such as CRISPR/Cas9 editing, transgenic overexpression, or knockdown studies in grass carp. Among these, *usf1* was downregulated in the intestine and kidney of HT fish. Previous studies have demonstrated that USF1 is a known regulator of metabolic genes [49], and plays important roles in response to hypoxia [50,51,52]. Jiang et al. found that increased protein levels and DNA binding of USF1 mediate the inhibition of CYP19 gene expression in the human placenta by hypoxia and Mash-2 [51,52]. In other studies of fish undergoing hypoxia stress, the expression or methylation changes in *usf1* were also detected [53,54]. The downregulation of *usf1* in this study may facilitate metabolic reprogramming under hypoxia. *trpv4* was downregulated in the brain of HT fish, which mediates calcium signaling and has been linked to oxygen sensing in vertebrates [55,56,57]. The alpha-2-macroglobulin (*a2m*), a high-molecular weight homotetrameric glycoprotein [58], was upregulated in the kidney of HT fish. In studies of vertebrates, A2M-AS1 is downregulated in cardiomyocytes and exerts protective effects against hypoxia/reoxygenation-induced cardiomyocyte injury by suppressing apoptosis and oxidative stress [59,60]. Functional validation of these genes in fish models is necessary to elucidate their precise roles in hypoxia tolerance.

## 5. Conclusions

This study integrated GWAS and multi-tissue transcriptome profiling to explore the genetic basis of hypoxia tolerance in grass carp. A total of 21 SNPs and 6 InDels associated with hypoxia tolerance were identified, with 2 SNPs on chromosome 10 and 13 reaching genome-wide significance. Candidate genes within these variants were enriched in pathways crucial for metabolic adaptation, including steroid biosynthesis and insulin signaling. Transcriptome analysis revealed distinct tissue-specific differential gene expression patterns under hypoxic stress, and DEGs were enriched in processes related to steroid biosynthesis, oxygen transport, and erythrocyte development. Integration of GWAS and transcriptome data highlighted 16 shared genes, notably *usf1* and *trpv4*, as key players in hypoxia tolerance. These findings provide valuable genomic resources and molecular markers for selective breeding programs aimed at enhancing hypoxia tolerance in grass carp. We will further validate the function of candidate genes and explore their potential application in molecular breeding programs.

## Figures and Tables

**Table 1:** The significant and suggestive SNPs associated with hypoxia tolerance in grass carp.

SNPs	Chromosome	Position	Allele	PVE (%)	*p* Value	Association
1	13	31,907,229	A/C	4.8	4.2568 × 10^−9^	significant
2	10	11,648,443	G/A	2.7	9.5401 × 10^−9^	significant
3	20	32,115,202	G/A	6.1	4.1755 × 10^−8^	suggestive
4	20	32,115,197	T/A	6.0	4.1763 × 10^−8^	suggestive
5	7	22,787,017	A/C	3.5	6.1038 × 10^−8^	suggestive
6	2	4,792,764	G/A	2.2	1.1057 × 10^−7^	suggestive
7	5	33,445,030	A/G	4.0	1.1164 × 10^−7^	suggestive
8	4	9,957,664	C/T	1.9	1.1423 × 10^−7^	suggestive
9	2	4,101,177	C/T	2.0	1.203 × 10^−7^	suggestive
10	7	22,787,042	T/C	3.3	1.2183 × 10^−7^	suggestive
11	10	36,269,337	A/G	5.2	1.268 × 10^−7^	suggestive
12	20	29,628,336	C/A	4.1	1.4681 × 10^−7^	suggestive
13	3	31,672,079	T/C	2.8	1.5794 × 10^−7^	suggestive
14	19	7,384,912	T/C	2.6	1.7027 × 10^−7^	suggestive
15	14	24,079,263	A/T	3.9	1.8668 × 10^−7^	suggestive
16	5	31,480,201	A/T	1.8	1.8728 × 10^−7^	suggestive
17	19	16,977,642	G/T	2.5	2.0578 × 10^−7^	suggestive
18	19	2,891,834	C/T	1.7	2.0833 × 10^−7^	suggestive
19	6	13,385	G/A	1.2	2.1928 × 10^−7^	suggestive
20	16	23,173,806	A/G	3.0	2.5773 × 10^−7^	suggestive
21	20	1,808,957	G/T	5.5	2.6181 × 10^−7^	suggestive

**Table 2:** Shared genes between the potential candidate genes identified by GWAS and DEGs obtained by RNA-seq analysis.

Gene Symbol	Chromosome	Position	Gene Annotation	Tissue Showing Differential Expression	Gene Regulation
*flo11*	20	29,675,108–29,692,533	flocculation protein FLO11	Brain	Down
*ncs1a*	5	31,515,563–31,537,983	neuronal calcium sensor 1a	Brain	Up
*myh*	5	31,409,911–31,463,184	myosin heavy chain, fast skeletal muscle-like	Brain	Down
*ipo13b*	20	29,625,934–29,674,126	importin 13b	Brain; Kidney	Up; Down
*tubb2*	2	4,049,561–4,054,417	tubulin, beta 2A class Iia	Brain	Down
*snx17*	20	1,844,003–1,958,074	sorting nexin-17	Brain	Up
*scara5*	20	1,781,417–1,809,123	scavenger receptor class A, member 5	Brain	Down
*trpv4*	5	33,489,408–33,514,568	transient receptor potential cation channel, subfamily V, member 4	Brain	Down
*usf1*	15	26,021,801–26,026,293	upstream transcription factor 1	Intestine; Kidney	Down; Down
*LOC127501978*	20	32,059,939–32,098,516	ncRNA	Intestine	Up
*dio3b*	20	32,118,645–32,121,485	iodothyronine deiodinase 3b	Intestine	Up
*uchl5*	10	8,839,438–8,845,469	ubiquitin carboxyl-terminal hydrolase L5	Intestine	Down
*tmod4*	16	23,160,790–23,168,927	tropomodulin 4	Kidney	Down
*a2m*	15	25,990,646–26,004,007	alpha-2-macroglobulin	Kidney	Up
*cratb*	19	7,391,651–7,439,445	carnitine O-acetyltransferase b	Liver	Up
*rgs13b*	10	8,824,407–8,827,219	regulator of G protein signaling 13b	Liver	Down

## Data Availability

The RNA-seq raw data have been submitted to the NCBI SRA under BioProject ID PRJNA1306682, which is accessible via the provided link: https://www.ncbi.nlm.nih.gov/bioproject/?term=PRJNA1306682, 15 August 2025. The raw genome resequencing data and phenotypic data may be made available upon request by contacting the corresponding author.

## Supplementary Materials

The following supporting information can be downloaded at: https://www.mdpi.com/article/10.3390/ani15243518/s1, Figure S1: Population structure analyses using InDels of grass carp. A. PCA analysis. B. Heatmap of genomic relatedness; Figure S2: Q-Q plots of grass carp subjected to hypoxia treatment. A. SNPs. B. InDels; Figure S3: The number of differentially expressed genes in the brain, intestine, kidney, liver, gill, and spleen between the HI and HT groups; Table S1: Primers used in the KASP analysis and qPCR experiments; Table S2: The candidate genes associated with significant and suggestive SNPs; Table S3: The candidate genes associated with suggestive InDels; Table S4: Summary of RNA-seq sequencing.
