# Supplementary material for: Integrative Genomics and Multi-Tissue Transcriptomics Identify Key Loci and Pathways for Hypoxia Tolerance in Grass Carp
Source: Animals (Basel). 2025 Dec 5;15(24):3518. doi: 10.3390/ani15243518 (PMC12729821; doi:10.3390/ani15243518)
Supplement: Supplementary file 1 [file animals-15-03518-s001.zip › animals-3975673-supplementary.pdf]

Article

Integrative Genomics and Multi-Tissue Transcriptomics Identify Key Loci and Pathways for Hypoxia Tolerance in Grass Carp

Wenwen Wang [1,2,†], Mengyang Chang [1,†], Suxu Tan [1,2], Yiming Hu [1,2], Xinlu Ren [1], Hongtao Xue [3], Lizheng Gao [1], Xiao Cao [4], Ya Wang [4], Qiyu Li [4] and Zhenxia Sha [1,2,5,*]

[1] Institute of Aquatic Biotechnology, College of Life Sciences, Qingdao University, Qingdao 266071, China
[2] Shandong Center of Technology Innovation for Biological Breeding of Premium Fish (Preparatory), Yantai 261418, China
[3] State Key Laboratory of Mariculture Biobreeding and Sustainable Goods, Yellow Sea Fisheries Research Institute, Chinese Academy of Fishery Sciences, Qingdao 266071, China
[4] Weishan County Nansihu Fishery Co., Ltd., Jining 277600, China
[5] Laboratory for Marine Fisheries Science and Food Production Processes, Qingdao Marine Science and Technology Center, Qingdao 266237, China
* Correspondence: shazhenxia@qdu.edu.cn
† These authors contributed equally to this work.

Supplemental tables and figures

Supplementary Table S1. Primers used in the KASP analysis and qPCR experiments.

Primer name	Sequence
SNP7-F1	GAAGGTGACCAAGTTCATGCTAGCAGCCGCTGATCTAAGCAAGGTA
SNP7-F2	GAAGGTCGGAGTCAACGGATTAGCAGCCGCTGATCTAAGCAAGGTG
SNP7-R	GAAGAACATCAACCAAAAACAGGAAACTC
SNP11-F1	GAAGGTGACCAAGTTCATGCTTGACCAAGAAAATCTGAACTGCGAA
SNP11-F2	GAAGGTCGGAGTCAACGGATTTGACCAAGAAAATCTGAACTGCGAG
SNP11-R	AATAACGATAAATCAGTTCATCAGGGAGG

Supplementary Table S2. The candidate genes associated with significant and suggestive SNPs.

Chromo-some	Gene name	Gene ID	Position	+/-	Gene description
2	clstn2a	127504555	4586021-4840396	-	calsyntenin 2a
2	tubb2	127502683	4049561-4054417	-	tubulin, beta 2A class Iia
2	LOC127522637	127522637	4149153-4153847	-	interferon-induced protein 44-like
2	dspa	127522625	4118547-4144978	+	desmoplakin a
2	slc22a23	127498540	4057167-4105635	-	solute carrier family 22 member 23
3	LOC127509639	127509639	31626736-31629122	-	uncharacterized LOC127509639
3	LOC127509627	127509627	31645387-31646011	-	uncharacterized LOC127509627
3	LOC127509653	127509653	31659125-31661691	-	uncharacterized LOC127509653
3	LOC127509642	127509642	31679802-31718739	-	uncharacterized LOC127509642
3	LOC127509622	127509622	31702413-31703915	-	uncharacterized LOC127509622
3	LOC127509640	127509640	31662233-31823087	+	uncharacterized LOC127509640
4	cd163	127510405	9972440-9984810	+	scavenger receptor cysteine-rich type 1 protein M130-like
4	syt10	127511484	9997730-10016696,	-	synaptotagmin X
4	cd163	127510393	9790637-9940504	+	scavenger receptor cysteine-rich type 1 protein M130-like
4	dmbt1	127510388	9817761-9921566	+	deleted in malignant brain tumors 1 protein-like
4	dmbt1	127511434	9940391-9970391	+	deleted in malignant brain tumors 1 protein-like
4	LOC127510412	127510412	9956067-9978172	+	uncharacterized LOC127510412
5	ankrd13a	127513695	33425544-33438292	-	ankyrin repeat domain 13A
5	gltpa	127513698	33482486-33488766	+	glycolipid transfer protein a
5	slc6a4b	127513588	33405184-33418678	+	solute carrier family 6 member 4b
5	git2a	127513699	33438581-33470866	+	G protein-coupled receptor kinase interacting Arf-GAP 2a
5	tchp	127513697	33472054-33480933	-	trichoplein, keratin filament binding
5	trpv4	127513360	33489408-33514568	+	transient receptor potential cation channel, subfamily V, member 4
5	hspb15	127512431	31463339-31466203	-	heat shock protein, alpha-crystallin-related b15
5	c9	127513324	31479167-31486227	+	complement component 9
5	gas1b	127513594	31490617-31492791	-	growth arrest-specific 1b
5	dab2	127512406	31466293-31477346	-	DAB adaptor protein 2
5	fbxw2	127513190	31505033-31512040	-	F-box and WD repeat domain containing 2
5	ncs1a	127513424	31515563-31537983	-	neuronal calcium sensor 1a
5	LOC127512887	127512887	31409911-31463184	-	uncharacterized LOC127512887
6	LOC127514919	127514919	2954-24645	-	uncharacterized LOC127514919
7	bola2	127515693	22758822-22765383	+	zgc:112271 protein
7	LOC127515694	127515694	22762057-22765009	-	uncharacterized LOC127515694
7	znf570	127515687	22768352-22770790	-	zinc finger protein 570-like
7	trnah-gug	127516898	22822727-22822798	-	transfer RNA histidin (anticodon GUG)
7	trnah-gug-2	127516899	22823228-22823299	-	transfer RNA histidin (anticodon GUG)
7	slx1b	127515691	22757355-22761541	-	SLX1 homolog B, structure-specific endonuclease subunit
7	zdhhc21	127515690	22771560-22794742	+	zinc finger DHHC-type palmitoyltransferase 21
7	nfib	127515682	22811044-22912109	+	nuclear factor 1 B-type-like

7	sema4f	127515680	22673575-22747563	+	sema domain, immunoglobulin domain (Ig), trans-membrane domain (TM) and short cytoplasmic do-main, (semaphorin) 4F
10	LOC127520894	127520894	11628239-11629755	-	granzyme B(G,H)-like
10	LOC127520891	127520891	11642589-11656194	-	mast cell protease 1A-like
10	fgf22	127520792	11672435-11698257	-	fibroblast growth factor 22
10	LOC127520244	127520244	11656389-11668153	-	granzyme B(G,H)-like
10	LOC127520889	127520889	11486505-11649628	-	mast cell protease 1A-like
10	LOC127520785	127520785	36191969-36296180	-	protocadherin-16-like
13	LOC127524632	127524632	31931043-31933899	-	plectin-like
13	dst	127524630	31841102-32038355	-	dystonin
14	LOC127494255	127494255	24089275-24100484	-	protocadherin beta-7-like
14	LOC127494804	127494804	24102459-24116947	-	protocadherin alpha-C2-like
14	LOC127494256	127494256	24111467-24114246	-	protocadherin alpha-C2-like
14	LOC127494257	127494257	24119577-24122966	-	protocadherin alpha-C2-like
14	LOC127494258	127494258	24097910-24108490	-	protocadherin alpha-C2-like
14	LOC127525752	127525752	23935708-24074848	-	protocadherin alpha-C2-like
16	lysmd1	127497847	23153163-23155418	-	LysM, putative peptidoglycan-binding, domain containing 1
16	scnm1	127497845	23155892-23159530	+	sodium channel modifier 1
16	ensab	127497850	23201118-23206789	-	endosulfine alpha b
16	LOC127497772	127497772	23116239-23141547	-	uncharacterized LOC127497772
16	LOC127497771	127497771	23130998-23143209	+	soluble guanylate cyclase 88E-like
16	tnfaip8l2b	127497849	23144341-23152826	+	tumor necrosis factor, alpha-induced protein 8-like 2b
16	tmod4	127497844	23160790-23168927	-	tropomodulin 4 (muscle)
16	vps72b	127497842	23174260-23197707	-	vacuolar protein sorting 72 homolog b
16	hormad1	127497843	23206843-23216463	+	HORMA domain containing 1
16	mcl1b	127497846	23198761-23200141	-	MCL1 apoptosis regulator, BCL2 family member b
19	LOC127501110	127501110	7366299-7375227	-	uncharacterized LOC127501110
19	cratb	127501077	7391651-7439445	+	carnitine O-acetyltransferase b
19	LOC127501088	127501088	7116870-7357424	-	SH3 and cysteine-rich domain-containing protein 2-like
19	il6	127501686	16985425-16988856	+	interleukin 6 (interferon, beta 2)
19	tomm7	127501687	16988741-16997761	-	translocase of outer mitochondrial membrane 7 homolog
19	hycc1	127501682	16998414-17037627	-	hyccin PI4KA lipid kinase complex subunit 1
19	rapgef5a	127501681	16900214-16971807	-	Rap guanine nucleotide exchange factor (GEF) 5a
20	dio3b	127501967	32118645-32121485	+	iodothyronine deiodinase 3b
20	LOC127501978	127501978	32059939-32098516	-	uncharacterized LOC127501978
20	LOC127501982	127501982	32121966-32144555	-	uncharacterized LOC127501982
20	ppp2r5cb	127501929	32160109-32191506	-	protein phosphatase 2, regulatory subunit B', gamma b
20	dio3b	127501967	32118645-32121485	+	iodothyronine deiodinase 3b
20	LOC127501978	127501978	32059939-32098516	-	uncharacterized LOC127501978
20	LOC127501982	127501982	32121966-32144555	-	uncharacterized LOC127501982
20	ppp2r5cb	127501929	32160109-32191506	-	protein phosphatase 2, regulatory subunit B', gamma b
20	LOC127502396	127502396	29572710-29621705	-	protein eva-1 homolog A
20	ipo13b	127502122	29625934-29674126	-	importin 13b
20	LOC127502117	127502117	29675108-29692533	+	uncharacterized LOC127502117
20	trnal-cag-29	127503121	1816290-1816372	+	transfer RNA leucine (anticodon CAG)
20	atraid	127502241	1828907-1832886	-	all-trans retinoic acid-induced differentiation factor
20	tab2	127502214	1747712-1777338	+	TGF-beta activated kinase 1 (MAP3K7) binding protein 2
20	scara5	127502226	1781417-1809123	-	scavenger receptor class A, member 5 (putative)
20	eif2b4	127502222	1815645-1827603	+	eukaryotic translation initiation factor 2B, subunit 4 delta
20	LOC127502251	127502251	1844003-1958074	-	uncharacterized LOC127502251
20	snx17	127502230	1833956-1874567	+	sorting nexin 17

Supplementary Table S3. The candidate genes associated with suggestive InDels.

Chromosome	Gene name	Gene ID	Position	+/-	Gene description
1	LOC127518917	127518917	15590954-15593067	-	uncharacterized LOC127518917
1	LOC127518838	127518838	15622674-15627104	+	uncharacterized LOC127518838
1	LOC127518759	127518759	15630115-15711865	+	uncharacterized LOC127518759
1	LOC127518840	127518840	15652241-15668048	+	uncharacterized LOC127518840
1	LOC127518776	127518776	15461893-15608974	+	uncharacterized LOC127518776
2	hs6st1a	127524613	1767700-1904553	+	heparan sulfate 6-O-sulfotransferase 1a
3	zgc:112038	127508419	23678122-23683559	-	uncharacterized protein LOC553673 homolog
3	LOC127508420	127508420	23710438-23723877	-	trypsin-like
3	LOC127509280	127509280	23727990-23730638	+	uncharacterized LOC127509280
3	cdipt	127508619	23758107-23763296	+	CDP-diacylglycerol--inositol 3-phosphatidyltransferase (phosphatidylinositol synthase)
3	zgc:123295	127508418	23684243-23695391	-	uncharacterized protein LOC641564 homolog
3	taok2a	127508608	23731558-23757067	+	TAO kinase 2a
3	LOC127508609	127508609	23763759-23777592	+	myc-associated zinc finger protein
3	nlk1	127508611	23778772-23791093	+	nemo-like kinase%2C type 1
10	uts2d	127521490	8810261-8812236	+	urotensin 2 domain containing
10	si:ch211-117l17.6	127520868	8817474-8820626	+	regulator of G-protein signaling 21

10	rgs13b	127520870	8824407-8827219	+	regulator of G protein signaling 13b
10	rgs2	127521624	8831662-8834538	+	regulator of G protein signaling 2
10	uchl5	127521446	8839438-8845469	-	ubiquitin carboxyl-terminal hydrolase L5
10	LOC127520867	127520867	8812843-8816479	+	platelet glycoprotein V
10	glrx2	127521447	8846052-8848962	-	glutaredoxin 2
10	b3galt2	127521389	8874560-8912481	-	UDP-Gal:betaGlcNAc beta 1%2C3-galactosyltransferase%2C polypeptide 2
11	LOC127522313	127522313	3585630-3587450	+	uncharacterized LOC127522313
11	LOC127522312	127522312	3603695-3641929	+	uncharacterized LOC127522312
11	lrp1aa	127522311	3499564-3676747	-	low density lipoprotein receptor-related protein 1Aa
15	LOC127495875	127495875	26010628-26012934	+	uncharacterized LOC127495875
15	LOC127496151	127496151	26013334-26017779	+	uncharacterized LOC127496151
15	usf1	127495004	26021801-26026293	+	upstream transcription factor 1
15	LOC127495323	127495323	25990646-26004007	-	alpha-2-macroglobulin
15	gkup	127495003	26028681-26038385	+	glucuronokinase with putative uridyl pyrophosphorylase
15	ubash3bb	127495002	26038686-26062455	+	ubiquitin associated and SH3 domain containing Bb
15	LOC127495007	127495007	26065037-26067100	+	uncharacterized LOC127495007
15	LOC127495006	127495006	26070106-26190958	+	uncharacterized LOC127495006
15	LOC127495005	127495005	26078840-26097507	-	uncharacterized LOC127495005

Supplementary Table S4. Summary of RNA-seq sequencing.

Sample	Raw reads	Clean reads	Clean base	Q20 (%)	Q30 (%)	GC (%)	Total mapping rate (%)
HI_Gi1	48,116,306	46,677,386	7.00	97.55	93.24	42.42	91.98
HI_Gi2	54,823,612	53,026,142	7.95	97.44	93.06	42.41	92.00
HI_Gi3	47,195,696	43,528,216	6.53	97.88	94.00	41.72	91.97
HI_In1	46,427,814	44,830,834	6.72	97.16	92.44	42.64	92.01
HI_In3	74,669,060	72,354,680	10.85	97.50	93.09	42.79	91.99
HI_In5	63,451,436	61,389,916	9.21	97.48	93.11	43.10	92.02
HI_Li1	42,649,688	41,496,240	6.22	97.25	92.64	42.64	91.95
HI_Li2	49,211,962	47,479,788	7.12	97.37	92.90	43.06	91.96
HI_Li3	46,956,354	45,424,242	6.81	97.36	92.85	42.59	91.94
HI_Sp1	61,057,288	58,953,584	8.84	97.49	93.12	42.20	92.03
HI_Sp2	66,407,832	63,623,668	9.54	97.56	93.27	42.23	91.99
HI_Sp3	46,683,696	44,798,054	6.72	97.58	93.31	42.99	92.00
HI_Br1	43,343,552	40,614,056	6.09	96.95	91.86	41.95	91.98
HI_Br2	42,759,160	39,911,646	5.99	97.72	93.50	42.00	91.97
HI_Br4	48,001,472	44,106,082	6.62	97.75	93.78	41.12	91.96
HI_Ki1	52,301,728	50,812,643	7.60	97.60	93.20	42.50	91.95
HI_Ki2	49,817,542	48,203,958	7.20	97.70	93.40	42.30	92.00
HI_Ki3	55,097,865	53,499,102	8.00	97.50	93.00	42.60	92.02
HT_Gi1	40,392,424	37,635,030	5.65	97.68	93.41	42.61	92.04
HT_Gi2	42,981,988	40,391,632	6.06	97.61	93.27	42.64	92.01
HT_Gi3	42,018,134	39,357,066	5.90	97.50	93.07	42.92	91.95
HT_In1	47,829,420	45,611,806	6.79	97.45	93.00	42.37	91.93
HT_In2	48,321,700	46,221,134	6.85	97.49	93.05	42.48	91.98
HT_In3	49,116,288	46,913,144	6.97	97.53	93.08	42.60	91.99
HT_Li1	47,204,136	45,187,902	6.70	97.48	93.01	42.45	91.97
HT_Li2	49,834,570	47,619,290	7.05	97.51	93.12	42.55	92.02
HT_Li3	46,503,842	44,509,612	6.65	97.47	93.00	42.30	91.96
HT_Sp1	50,108,712	47,980,198	7.10	97.50	93.10	42.41	92.00
HT_Sp2	48,990,160	46,771,804	6.95	97.46	92.95	42.36	91.95
HT_Sp3	47,719,034	45,492,922	6.81	97.44	92.88	42.38	91.94
HT_Br1	48,662,176	46,415,002	6.91	97.55	93.02	42.49	91.99
HT_Br2	49,371,026	47,190,430	7.00	97.56	93.11	42.52	91.98
HT_Br3	48,245,984	46,089,398	6.83	97.52	93.00	42.40	91.97
HT_Ki1	55,007,439	53,104,227	7.50	97.40	93.05	43.00	92.03
HT_Ki2	43,343,552	40,614,056	6.09	96.95	91.86	41.95	91.98
HT_Ki3	46,759,160	42,911,646	6.39	97.72	93.50	42.00	91.97

HI: hypoxia-intolerant; HT: hypoxia-tolerant; Gi: gill; In: intestine; Li: liver; Sp: spleen; Br: brain; Ki: kidney.

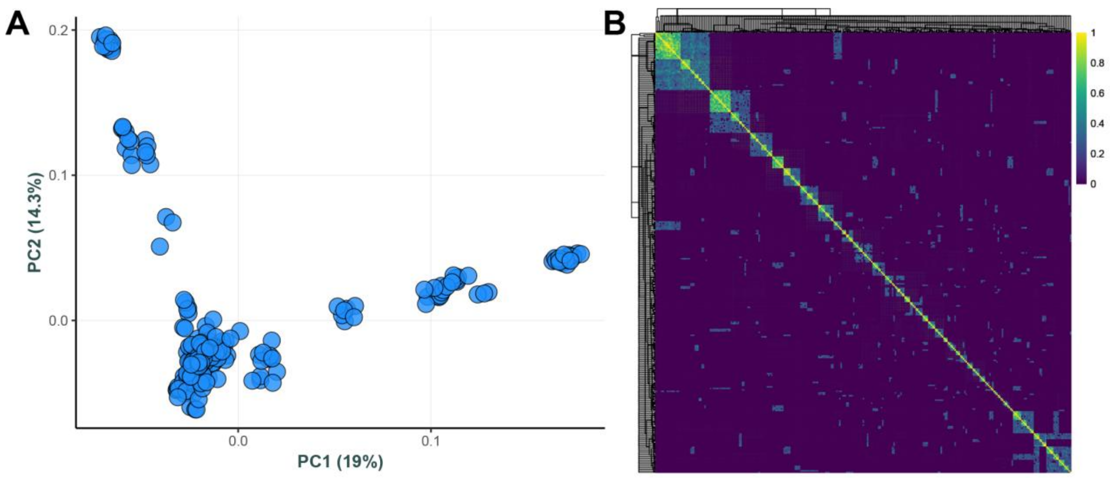

Supplementary Figure S1. Population structure analyses using InDels of grass carp. (**A**) PCA analysis. (**B**) Heatmap of genomic relatedness.

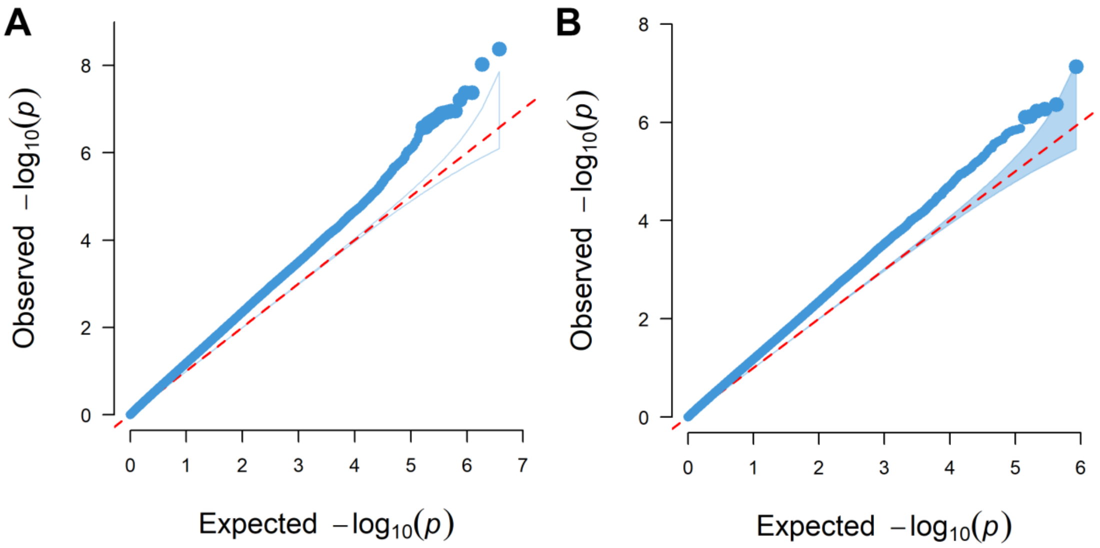

Supplementary Figure S2. Q-Q plots of grass carp subjected to hypoxia treatment. (**A**) SNPs. (**B**) InDels.

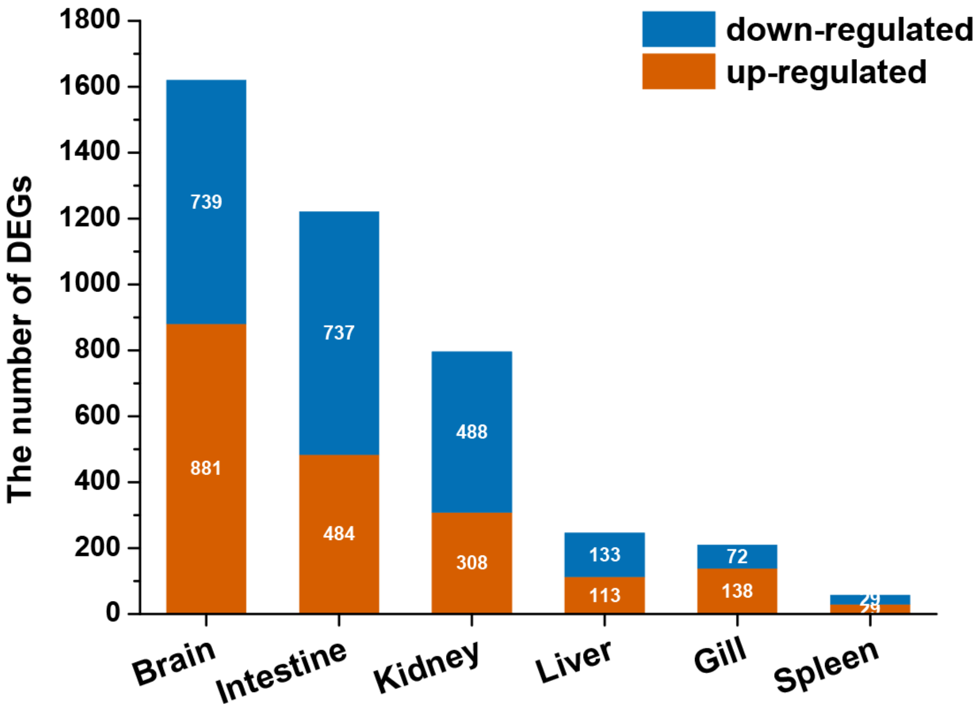

Supplementary Figure S3. The number of differentially expressed genes in the brain, intestine, kidney, liver, gill, and spleen between the HI and HT groups.
